# Supplementary material for: In Vivo Induction of Leukemia-Specific Adaptive and Innate Immune Cells by Treatment of AML-Diseased Rats and Therapy-Refractory AML Patients with Blast Modulating Response Modifiers
Source: Int J Mol Sci. 2024 Dec 16;25(24):13469. doi: 10.3390/ijms252413469 (PMC11679655; doi:10.3390/ijms252413469)

Supplementary Materials for

In vivo Induction of Leukemia Specific Adaptive and Innate Immune Cells By Treatment of AML-Diseased Rats and Therapy-Refractory AML-Patients with Blast Modulating Response Modifiers

M. Atzler[1,2], T. Baudrexler [1,2,†], D. C. Amberger [1,3,†], N. Rogers [1,2], A. Rabe [1,2], J. Schmohl [4], R. Wang [1], A. Rank [2,5],
O. Schutti [1,4] K. Hirschbuehl[2,5], M. Inngjerdingen [6], D. Deen [1,2], B. Eiz-Vesper [7], C. Schmid [2,5,‡]
and H. M. Schmetzer [1,2,‡]

Table S1. Subtypes of leukemic blasts, DC and DCleu, adaptive and innate immunity cell subsets in rats' blood.

		Name of Subgroups	Surface Marker	Referred to	Abbreviation	Reference
	**Blast cells**	Leukemic blasts	RM124+	WB	Bla/WB	[30]
		Proliferating blasts	Bla+Ki67+	WB	Bla_{prol}/WB	[30]
	**Dendritic cells**	Dendritic cells	DC e.g. CD86+, CD103+	WB	DC/WB	[12]
		Leukaemia derived DC	DC+Bla+	WB	DC_{leu}/WB	[12]
		MHC II	MHC II	WB	MHC+Bla/WB	[30,12]
**Adaptive Immune system**	**T lymphocytes**	CD3+ pan T cells	CD3+	WB	CD3+/WB	[15]
		CD4+ coexpressing T cells	CD3+CD4+	CD3+	CD4+CD3+/CD3+	[15]
		CD8+ coexpressing T cells	CD3+CD8+	CD3+	CD8+CD4+/CD3+	[15]
		Memory like T cells	CD3+CD4+CD62L++<br>CD3+CD4-CD62L++	CD3+<br>CD3+	CD4+T_{mem}/CD3+<br>CD8+T_{mem}/CD3+	[31]
		Regulatory T cells	CD3+CD4+Foxp3+CD25+	CD3+	CD4+T_{reg}/CD3+	[31]
**Innate Immune system**	**Natural killer cells**	CD3-CD56+ NK cells	NKR-P1A+ CD3-	lymphocytes	NKcell/cells	[29]
	**Natural killer T cells**	6B11+ NKT cells	NKR-P1A+ CD3+	lymphocytes	NKTcell/cells	[29]

WB whole blood; MHC II major histocompatibility complex type II.

Table S2. Subtypes of leukemic blasts, DC and DC_{leu}, adaptive and innate immunity and leukemia specific cell subsets in human blood samples.

	Name of Subgroups	Surface Marker	Referred to	Abbreviation	Reference
**Blast cells**	Blasts	Bla e.g. CD34+, CD117+	WB	Bla/WB	[12]
	Proliferating blasts	Bla+DC-CD71+	Bla	Bla_{prol71}/Bla	[12]
	Proliferating blasts	Bla+DC-IPO38+	Bla	$Bla_{prolIPO38}$/<br>Bla	[12]
**Dendritic cells**	Dendritic cells	DC+ e.g. CD80+, CD206+	WB,	DC/WB	[12]
	Leukaemia derived DC	DC+Bla+	WB DC, blasts	DC_{leu}/WB<br>DC_{leu}/DC<br>DC_{leu}/blasts	[12]
	Mature migratory DC	DC+CD197+	WB	DC_{mat}/WB	[16]
	Mature migratory DC_{leu}	DC+Bla+CD197+	WB	$DC_{leu\text{-}mat}$/WB	[16]
**B lymphocytes**	CD19+ memory B cells	CD19+CD27+<br>IgD+	CD19+	$Bcell_{memory}$/<br>CD19+	[31]
**T lymphocytes**	CD3+ pan T cells	CD3+	lymphocytes	CD3+/cells	[15]
	CD4+ T cells	CD3+CD4+	CD3+	T_{CD4+}/CD3+ or<br>CD3+CD4+/CD3+	[15]
	CD4- T cells	CD3+CD4-	CD3+	T_{CD4-}/CD3+ or<br>CD3+CD4-/CD3+	[15]
	T helper cells 1<br>T helper cells 17	CCR4-CXCR3+CCR5+<br>CCR6-<br>CCR4+CXCR3-CCR5-<br>CCR6+	CD4+<br>CD4+	TH_1+/CD4+<br>TH_{17}+/CD4+	[31]

**Adaptive immune system**		Non-naive T cells	CD3+CD45RO+<br>CD3+CD45RO+CD4+<br>CD3+CD45RO+CD4-	CD3+<br>T_{CD4+}<br>T_{CD4-}	$T_{non-naive}$/ CD3+<br>$T_{non-naïve\ CD4+}$/ T_{CD4+}<br>$T_{non-naïve\ CD4-}$/T_{CD4-}	[16,22]
		Central (memory) T cells	CD3+CD45RO+CD197+<br>CD3+CD45RO+CD197+CD4+	CD3+<br>T_{CD4+}	T_{cm}/CD3+<br>$T_{cm\ CD4+}$/T_{CD4+}	[16,22]
		Effecor (memory) T cells	CD3+CD45RO+CD197-<br>CD3+CD45RO+CD197-CD4+	CD3+<br>T_{CD4+}	T_{em}/CD3+<br>$T_{em\ CD4+}$/T_{CD4+}	[16,22]
		Proliferating T cells - early	CD3+CD69+<br>CD3+CD4-CD69+	CD3+<br>T_{CD4-}	$T_{prolCD69+}$/ CD3+<br>$T_{prolCD4-CD69+}$/T_{CD4-}	[16,22]
		Proliferating T cells - late	CD3+CD71+	CD3+	$T_{prolCD71+}$/ CD3+	[16,22]
**Innate immune system**	**Cytokine induced killer cells**	CD3+CD56+ CIK cells	CD3+CD56+	lymphocytes	CIKcell/cells	[15]
	**Natural killer cells**	CD3-CD56+ NK cells	CD3-CD56+	lymphocytes	NKcell/cells	[15]
	**Invariant natural killer T cells**	6B11+ iNKT cells	6B11+	lymphocytes	iNKTcell/cells	[32]
	**LEUKEMIA SPECIFIC CELLS**					
**Adaptive immune system**	**T lymphocyte cells ***	CD4+ coexpressing T $cells_{leu}$	CD3+CD4+IFNy+	$T_{CD4+leu}$	$T_{CD4+leu}$/T_{CD4+}	[32,22]
		CD8+ coexpressing T $cells_{leu}$	CD3+CD4-IFNy+	$T_{CD4-leu}$	$T_{CD4-leu}$/T_{CD4-}	[32,22]
**Innate immune system**	**Cytokine induced killer cells ***	CD3+CD56+ CIK $cells_{leu}$	CD3+CD56+IFNy+	CIKcell	$CIKcell_{leu}$/ CIKcell	[32,22]
	**Invariant natural killer T cells ***	6B11+ iNKT $cells_{leu}$	6B11+IFNy+	iNKTcell	$iNKTcell_{leu}$/ iNKTcell	[32,22]

* Evaluated by cytokine secretion assay (P1482/P1511) and by intracellular cytokine assay (P1601) +LAA stimulation

Table S3. Treatment regime for P1482 and P1601 using Kit M (leukine (GM-CSF) and Prostavasin (PGE1). Courses of the disease are given in Figure 7 and 8**.**

a) P1482

**Day**	**Drug**	**Dosage (iv)**	**Schedule**
**11***	GM-CSF	50 μg/m²/4 hours	8-12 am
	PGE_1	20 μg (in total)	1-3 pm
**12**	PGE_1	20 μg (in total)	8-10 am
	GM-CSF	50 μg/m²/4 hours	11 am-3 pm
	PGE_1	20 μg (in total)	8-10 pm
**13**	PGE_1	20 μg (in total)	8-10 am
	GM-CSF	50 μg/m²/4 hours	11 am-3 pm
	PGE_1	20 μg (in total)	8-10 pm
**14**	PGE_1	20 μg (in total)	8-10 am
	GM-CSF	75 μg/m²/4 hours	11 am-3 pm
	PGE_1	40 μg (in total)	8-10 pm
**15**	PGE_1	40 μg (in total)	8-10 am
	GM-CSF	75 μg/m²/4 hours	11 am-3 pm
	PGE_1	40 μg (in total)	8-10 pm
**16-38**		Percede as day 15	

b) P1601			
**Day**	**Drugs**	**Dosage (iv)**	**Schedule**
**9***	GM-CSF	50 μg/m²/4 hours (77.5 μg in total)	8-12 am
	PGE_1	20 μg (in total)	1-3 pm
**10**	PGE_1	20 μg (in total)	8-10 am
	GM-CSF	50 μg/m²/4 hours (77.5 ug in total)	11 am- 3 pm
	PGE_1	20 μg (in total)	6-8 pm
**11**	PGE_1	20 μg (in total)	8-10 am
	GM-CSF	50 ug/m²/4 hours (77.5 μg in total)	11 am- 3 pm
	PGE_1	20 μg (in total)	6-8 pm
**12**	PGE_1	20 μg (in total)	8-10 am
	GM-CSF	75 μg/m²/4 hours (116 μg in total)	11 am- 3 pm
	PGE_1	40 μg (in total)	6-8 pm
**13**	PGE_1	40μg (in total)	8-10 am
	GM-CSF	75 μg/m²/4 hours	11 am- 3pm
	PGE_1	40 μg (in total)	6-8 pm
**14**	PGE_1	40 μg (in total)	8-10 am
	GM-CSF	75 μg/m²/4 hours	10 am- 12 pm
	PGE_1	40 μg (in total)	12- 1 pm
**15-26**		Proceded as day 14	

*Observation time and monitoring of blasts start at Day 1; Kit M treatment starts at Day 11 in patient 1482, respectively day 9 in patient 1601

Supplementary figures for the gating strategy of (leukemia specific) immune cells via Flowcytometry:

-Gating of all cells was done using antibody combinations as given in ***Table 3 (supplement)***. This was true for rat as well as for human ex vivo and in vivo monitorings.

-Gating of rat samples was done in Oslo by members of the lab of Prof. Rolstad

-Gating and monitoring for human cell monitoring of patients' samples of treated patients from Diakonieklinikum Stuttgart and from University Hospital of Augsburg was performed in the 'FACS labs' Augsburg (Prof Rank) and the LMU Klinikum Großhadern in Munich according to:

Waidhauser et al [31]

1) Gating strategy for various immune subsets in gated lymphocytes:

a)…for T-, B-, NK-, CIK-cells

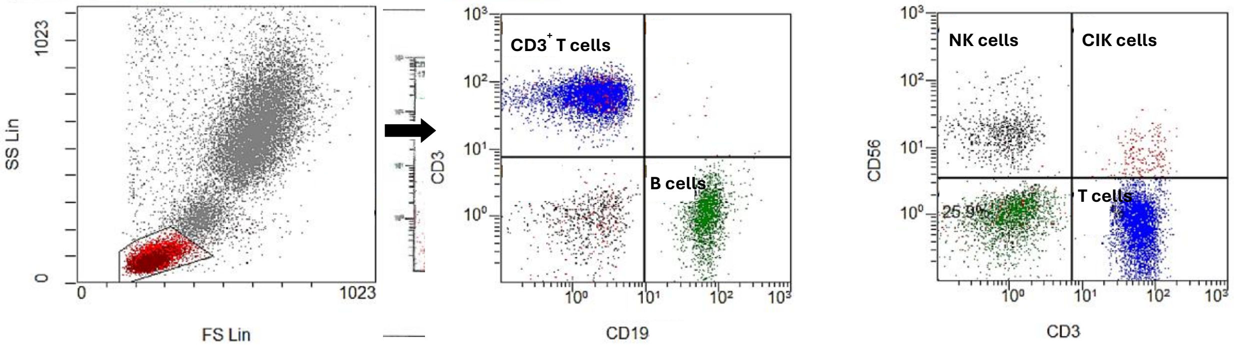

b)..for T cell subsets and proliferating subsets

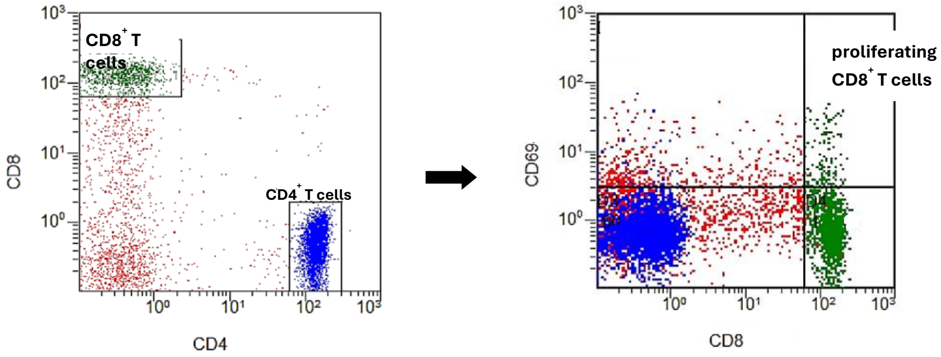

c)..for B cell subsets (Bmemory cells)

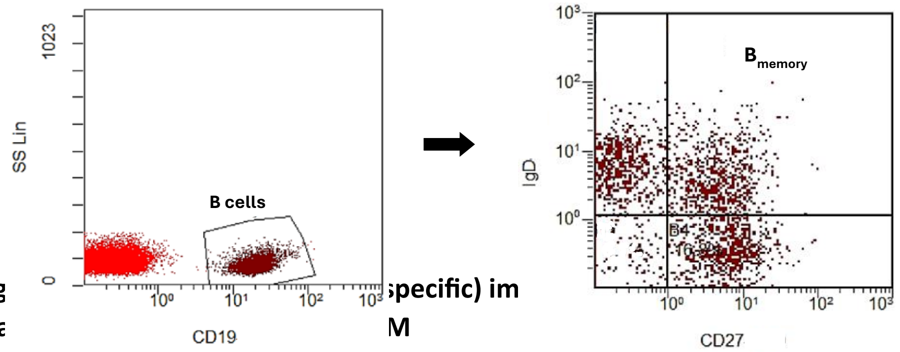

2) Gating [illegible] specific) im[illegible] vs under or after trea[illegible] M

a)…for (leukemia specific, IFNy producing) T cell subtypes (CD4+ and CD3+))

Before… **under/after Kit M therapy**

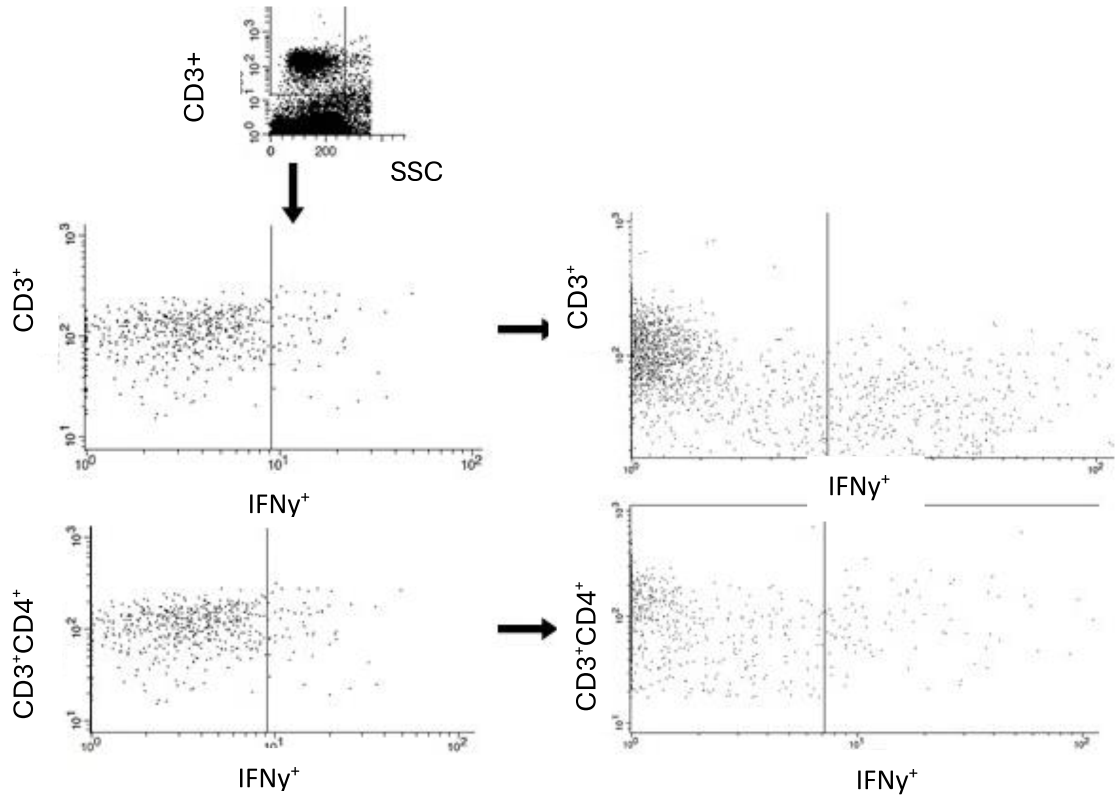

b)…for (leukemia specific, IFNy producing) NK cells

Before… **under/after Kit M therapy**

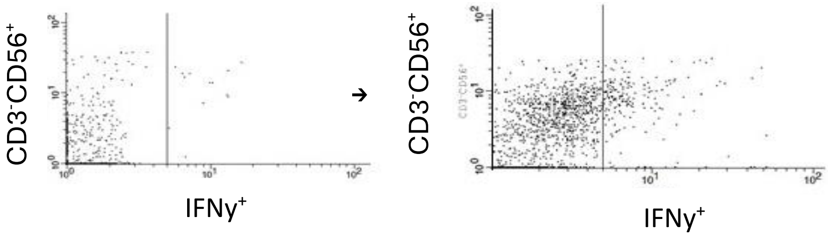

c) …for dendritic cells

Before… **under/after Kit M therapy**

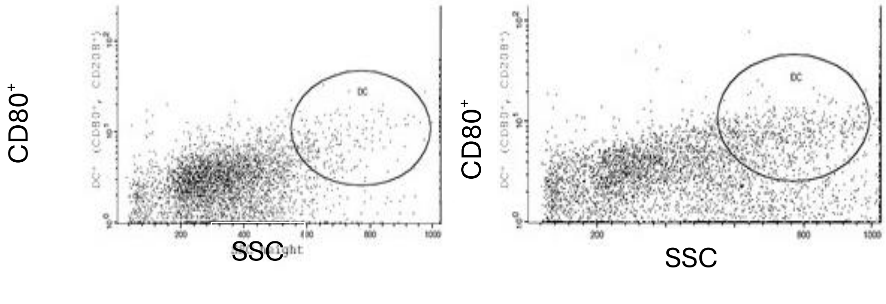

Supplement: Supplementary file 1 [file ijms-25-13469-s001.zip › ijms-3212139-supplementary.pdf]
